# Nitro‐Group π System Drives the Interaction of RRx‐001 with Electrons in Solution

**DOI:** 10.1002/chem.202500859

**Published:** 2025-05-09

**Authors:** Barbora Sedmidubská, Sergey Denisov, Mehran Mostafavi, Stephan Denifl, Fahrad Izadi, Milan Ončák, Thomas F.M. Luxford, David Chvátil, Jiří Pinkas, Jaroslav Kočišek

**Affiliations:** ^1^ J. Heyrovský Institute of Physical Chemistry of the CAS Dolejškova 3 Prague 182223 Czech Republic; ^2^ Institut de Chimie Physique UMR 8000 CNRS/Université Paris‐Saclay Bâtiment 349 Orsay 91405 France; ^3^ Department of Nuclear Chemistry Faculty of Nuclear Sciences and Physical Engineering Czech Technical University in Prague Břehová 7 Prague 11519 Czech Republic; ^4^ Universität Innsbruck Institut für Ionenphysik und Angewandte Physik Technikerstraße 25 Innsbruck 6020 Austria; ^5^ Center for Molecular Biosciences Innsbruck Universität Innsbruck Technikerstraße 25 Innsbruck A‐6020 Austria; ^6^ Department of Chemistry and Biochemistry Texas State University San Marcos TX 78666 USA; ^7^ Department of Accelerators Nuclear Physics Institute of the CAS Rez 25068 Czech Republic

**Keywords:** catalytic electron, electron attachment, low‐energy electrons, radiosensitizer, state selective, low‐energy electrons

## Abstract

Reactivity toward low‐energy electrons (LEE) has been hypothesized as a cause of radio‐modifying properties for various molecules. LEE's transient nature, however, prevents the establishment of clear links between initial processes at the sub‐ps time scale and the final products of radiolysis. Here, such links are explored for the radio‐modifying compound RRx‐001 (1‐bromoacetyl‐3,3‐dinitroazetidine).

Picosecond pulse radiolysis demonstrates the high scavenging capacity of the molecule for secondary quasi‐free and solvated electrons forming stable parent anions confirmed by studies of microsolvated RRx‐001 in clusters. The anions decay either via auto‐detachment of an electron or dissociate involving hydrogen transfer from solvent, resulting in NO_2_ and 1‐(bromoacetyl)‐3‐nitroazetidine. Surprisingly, no Br dissociation is observed despite its high electron affinity. We assign this behavior to the “inaccessibility” of sigma virtual states for electrons in the solvent, which can be of a general nature.

## Graphical abstract

The combination of cluster experiments, transient absorption spectroscopy, and NMR spectroscopy with theory enables tracking of the chemistry induced by secondary low‐energy electrons to RRx‐001 in solution from the initial interaction to the final radiolysis products. The catalytic and state‐selective nature of the LEE interaction is identified, with consequences for the understanding of the LEE action in radiation damage in general.

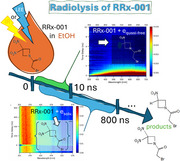

## 1 Introduction

Low‐energy electrons (LEE) in liquid environments have fascinated scientists for years due to their importance from the very fundamental processes to complex biological mechanisms.^[^ ^1^, ^2^, ^3^, ^4^ ^]^ LEE studies in realistic media are complicated by the transient nature of these species. When LEE are created in the environment, either directly or as a consequence of the slowing down an avalanche of fast secondary electrons, they rapidly interact with the neighboring molecules, inducing chemical changes or orienting the molecules into small cavities becoming solvated electrons.^[^ ^5^, ^6^ ^]^ To address the fastest initial processes, only a handful of ultrafast spectroscopy experiments exist with a limited range of possible reactant and solvent combinations.^[^ ^7^, ^8^, ^9^, ^10^, ^11^, ^12^, ^13^ ^]^ Despite the significant progress in recent years, dynamics of the LEE‐induced processes or simple interpretation of the experimental observations in bulk remain challenging.^[^ ^14^, ^15^ ^]^ Experiments with clusters, small aggregates of matter isolated in a vacuum, represent a possible solution, allowing for detailed exploration of reaction dynamics in a wide range of molecular systems as well as modeling of the electron scattering process.^[^ ^16^, ^17^ ^]^ Additionally, in clusters, the need for ultrafast spectroscopy required in bulk can be substituted by direct observation of the photoelectron as a probe,^[^ ^18^, ^19^ ^]^ or by the simple use of free ballistic electrons as a pump,^[^ ^20^, ^21^, ^22^ ^]^ selectively addressing the initial LEE interactions. The present study combines experiments addressing three different timescales and three different levels of complexity of the electron interaction with a promising cancer drug and radio‐modifier RRx‐001 to obtain a detailed understanding of its radiation chemistry with respect to processes induced by LEE. Interpretation supported by computational modeling allows us to follow the reactions from the initial phases when the single electron interacts with RRx‐001, forming transient negative ions through ps dynamics to final radiolysis products stable on long timescales (days).

The RRx‐001 is a derivative of the explosive TNAZ (C_3_H_4_N_4_O_6_, 1,3,3‐trinitroazetidine). Originally an aerospace molecule started to be studied for repurposing as an anticancer drug due to its activity and low toxicity shown in experimental models.^[^ ^23^ ^]^ The uniqueness of RRx‐001 lies in showing both normal tissue protection and antitumor cytotoxicity.^[^ ^24^ ^]^ RRx‐001 has already been successfully examined in clinical studies in Phase 3 and Phase 2.^[^ ^25^, ^26^ ^]^ Cellular trials show this molecule has the potential to be a stand‐alone cancer therapeutic as well as radiosensitizer.^[^ ^23^, ^27^ ^]^ The molecule possesses three highly electron‐affine groups: one bromine and two nitro groups with electron affinities of 3.36^[^ ^28^ ^]^ and 2.27 eV,^[^ ^29^ ^]^ respectively, which makes it a prototypical example of an electron affinic radiosensitizer.^[^ ^30^ ^]^

## 2 Results

Our first experiments focused on the scavenging of the LEE by RRx‐001 molecules in solution. The picosecond pulse radiolysis platform ELYSE enables the observation of the reaction kinetics of the molecules with electrons forming upon the initial radiolysis pulse. Particularly two types of secondary electrons, the quasi‐free electrons and solvated electrons.^[^ ^2^, ^31^ ^]^ The measurement is based on the detection of the intensity of the absorption band observed at 600 nm, characteristic for solvated electrons, as a function of the initial concentration of the RRx‐001 in the solution and time. The measurement was conducted in ethanol instead of water, while the second can be considered a more natural environment for the studied radiosensitizing compound. The main reasons are slower dynamics of the solvated electrons in ethanol and less competing electron scavenging reactions, particularly the well‐known OH^−^ formation channels in water.^[^ ^32^ ^]^ Consequently, the measurements in ethanol provide more reliable quantities for direct RRx‐001 interaction with low‐energy electrons. However, the lower complexity of the solution composition and chemistry, with respect to the biological medium, should be kept in mind. Characteristic constants for the interaction were evaluated. The decrease of the solvated electron signals in various concentrated solutions at a maximum around T = 2 ps after the radiolysis pulse is used to evaluate the C37 value, defined as a molar concentration of the substance needed to scavenge 63% of quasi‐free electrons (see Supporting Information for details). The C37 value for RRx‐001 is 0.2 M (room temperature, atmospheric pressure). We can compare that scavenging with other scavengers of the quasi‐free electrons. For example, NO_2_^−^ has C37 = 1.6 M,^[^ ^10^ ^]^ or for H^+^ C37 = 3.5 M. As we can observe, RRx‐001 has a very high scavenging capacity for quasi‐free electrons.

The reactivity of the RRx‐001 with solvated electrons can be evaluated from the change in the slope of the decay of the solvated electron absorption band at 600 nm (see Supporting Information). The obtained k_*abs*_ is equal to 9.4×10^9^ dm^3^mol^−1^s^−1^. This value is also high, comparable to some radiosensitizers (e.g. fluorouracil 1.2×10^10^ dm^3^mol^−1^s^−1^).^[^ ^32^ ^]^ The value is close to the value of the rate constant controlled by diffusion. That means there is no kinetic barrier for the reaction of the molecule with solvated electrons.^[^ ^33^ ^]^

The initial experiments at ELYSE platform show strong scavenging of quasi‐free as well as solvated electrons by RRx‐001. Further, we will explore what happens with the molecule upon the interaction.

### 2.1 Initial Electron Interaction Studied on Model Clusters

**Figure 1:**
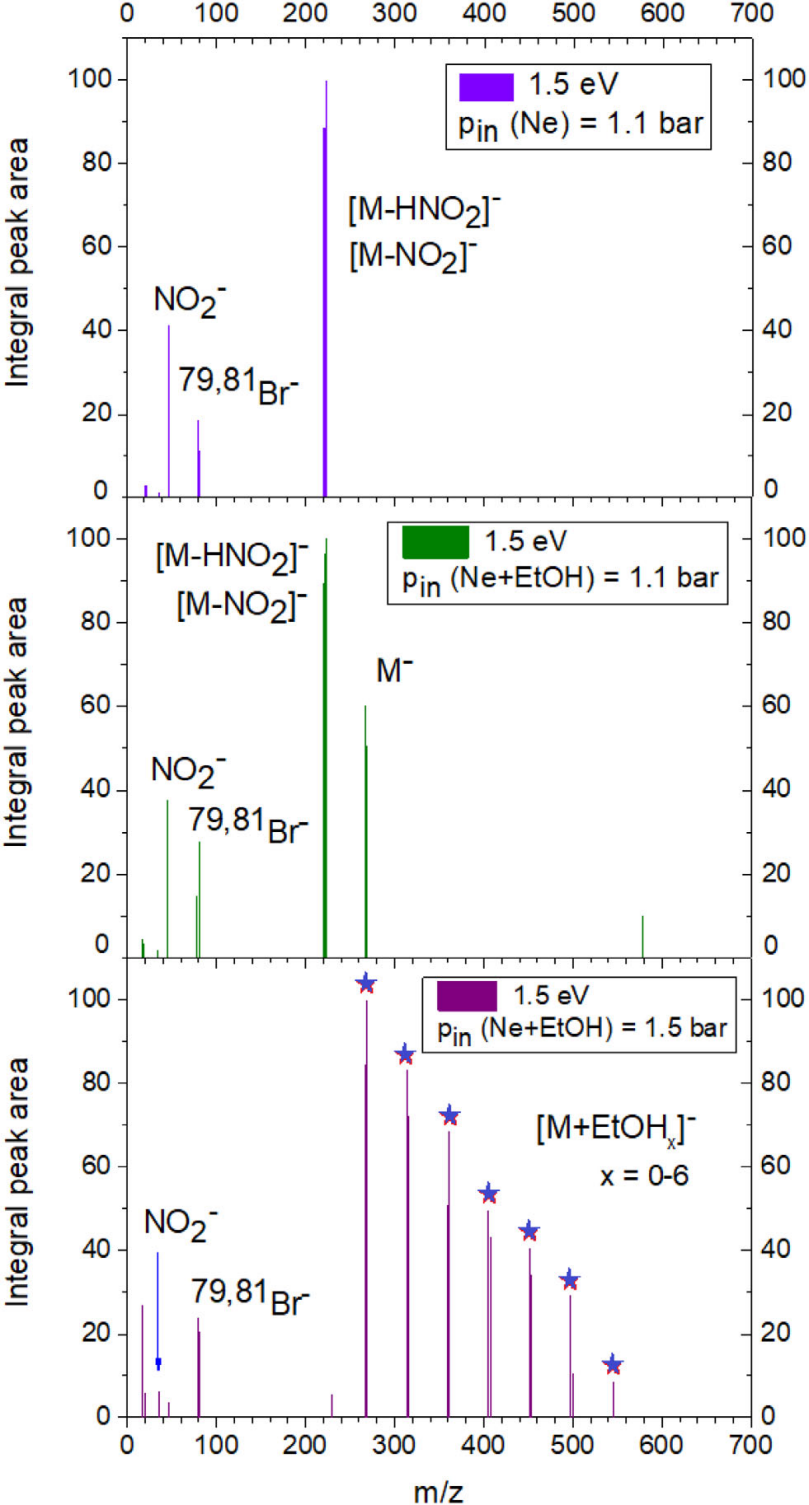
Mass spectra of negative ion fragments upon attachment of ballistic electrons to neutral mixed clusters of RRx‐001 with ethanol (RRx‐001)EtOH_*k*_ in molecular beam experiments. The spectra were measured at an incident electron energy of 1.5 eV, where the electron current is stabilized enough to provide reproducible results. The pressure of neon expansion gas is used to control the amount of ethanol EtOH_*k*_ in the precursor cluster from a) low through b) medium to c) high. We are presenting integrated intensities subtracted from the NO_2_ background signal, raw data are in Supporting Information together with equivalent spectra for RRx‐001 ‐ water clusters.

The initial step of the electron interaction can be modeled in experiments with ballistic (free) electrons interacting with microsolvated clusters, as demonstrated in several of our recent works (e.g., refs. [34, 35]). The mass spectra showing the evolution of the fragmentation of the RRx‐001 at different levels of solvation in ethanol upon collisions with free 1.5 eV electrons measured at CLUB experimental setup are shown in Figure 1. The different levels of neutral RRx‐001 precursor microsolvation in ethanol were achieved by changing the stagnation pressure of the neon carrier gas with constant ethanol intake via the nafion membrane as described in the methods section.

At the lowest solvation level, the most intense peaks can be assigned to the signal of the ions forming after the loss of NO_2_ and HNO_2_ neutrals, [M‐NO_2_]^−^ and [M‐HNO_2_]^−^, respectively. The mass spectrum is similar to the mass spectrum of the isolated molecule in our setup reported recently.^[^ ^36^ ^]^

At medium solvation conditions, we can already observe the parent anion and that the relative intensity for all dissociation channels decreases.

At the highest solvation conditions, the dissociation channels are significantly suppressed, only a small signal of Br^−^ and NO_2_^−^ is detected, and the spectrum is strongly dominated by parent cluster anions in the form RRx‐001(EtOH)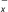. Interestingly, in the present spectra, measured at constant energy of electrons, the signal of [M‐HNO_2_]^−^, [M‐NO_2_]^−^ anions drop much faster than those of Br^−^ and NO_2_^−^ anions, despite of the fact that in the recent gas phase study, we concluded that a major part of the later ions come from the metastable decay of the further‐mentioned ones. It will be expected that energy transfer to solvent should result in the increase of the signal of [M‐HNO_2_]^−^, [M‐NO_2_]^−^ with respect to Br^−^ and NO_2_^−^. We assign this observation to the different states involved in the DEA as discussed in our study of isolated RRx‐001.^[^ ^36^ ^]^ The [M‐HNO_2_]^−^, [M‐NO_2_]^−^ anions are accessible only from the lowest‐lying state at very low energies of the electron, but the DEA channels resulting in Br^−^ and NO_2_^−^ are accessible also via σ* state at higher energies of incoming electrons. Since the internal energy of the precursor clusters is given by the expansion conditions, the energy available for dissociation or transfer to the solvent is given by the energy of the incoming electron.^[^ ^37^ ^]^ The narrow low‐energy resonance will be therefore closed first, while states accessible at higher electron energies will remain open. For better understanding of this interpretation, we direct the reader to our nimorazole paper^[^ ^21^ ^]^ where the described behavior is directly demonstrated on the energy‐dependent ion yields, since the energy difference between low‐ and high‐energy DEA channels is several eV. In the present experiments, we were not able to resolve individual resonances in the energy‐dependent ion yields (see Supporting Information).

For consistency with the pulse radiolysis experiment performed in ethanol, we present here the data for solvation in ethanol. However, we also performed a cluster study in water to demonstrate the general validity of the observed behavior. The spectra for microhydrated RRx‐001 clusters are shown in Figure S4. The only difference that we observed is that water forms clusters with RRx‐001 much better than ethanol. In other words, clusters with higher amounts of solvent molecules are formed at the same expansion conditions for water, when compared to ethanol.

The electron attachment experiments show that all DEA channels are closing with increasing levels of EtOH micro‐solvation due to the energy transfer to solvent and formation of M^−^ parent anions. We can conclude that these anions are formed with lifetimes longer than microseconds, as observed in our experiments. However, the electron attachment experiments cannot give us any information about neutral radiolysis products, these can be identified by absorption spectroscopy discussed further.

#### 2.1.1 Reaction Intermediates

**Figure 2:**
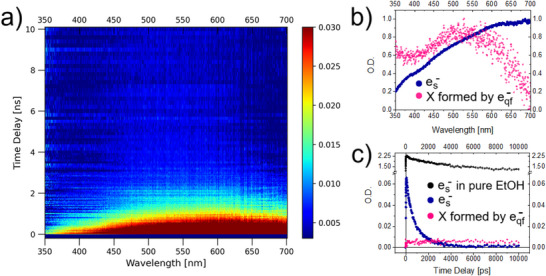
Data gained based on spectroscopic measurements of 70 mM solution of RRx‐001 in ethanol on ELYSE platform VD, in cuvette with circulation using picosecond pulse radiolysis. Panel a) shows 3D graph containing optical densities (O.D.) of forming transient species in irradiated solution in dependence on wavelength (nm) and time delay (ns). Panel b) is the absorption spectra and panel c) kinetics of two transient species in solution obtained by deconvolution of data on the left side using SKANA program.

**Figure 3:**
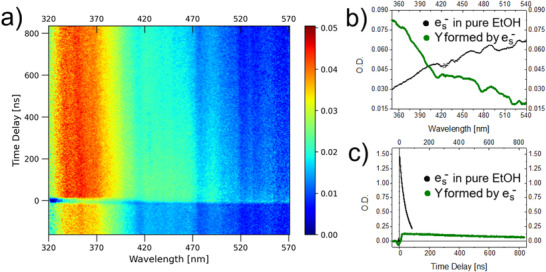
Data gained based on spectroscopic measurements of 70mM solution of RRx‐001 in ethanol on ELYSE platform VD2, in static cell using ns‐µs pulse radiolysis. Panel a) shows 3D graph containing O.D. of forming transient species in irradiated solution in dependence on wavelength (nm) and time delay (ns). Panel b) is the absorption spectra and panel c) kinetics of two transient species in solution obtained by deconvolution of data on the left side using SKANA program.

To identify the reaction intermediates in the process of RRx‐001 interaction with secondary electrons, we recorded 3D spectra at the ELYSE platform, measuring the absorbance of the RRx‐001 solution as a function of the wavelength and the time delay between the electron radiolysis pulse (pump) and the excitation light pulse (probe). Two spectra recorded on Vis–UV detection (VD) and Vis–UV detection 2 (VD2) lines of the ELYSE platform are depicted in Figures 2 and 3, respectively.

The raw data from the measurements of absorption spectra for 1 to 10 ns delay range of the 70 mM solution depicted in Figure 2 show a broad feature from 400 nm on, that can be assigned to the solvated electrons. This feature significantly complicates the detection of any absorption bands of reaction intermediates in this spectral region. However, information about the long‐lived species can be obtained based on the dynamical information provided by the delay measurement, since solvated electrons decay relatively fast, in approximately 2 ns, as better demonstrated in Figure 2c.

Already from the 3D spectra, we can see that after the complete decay of the solvated electrons, there is still a weak signal in the 450 to 650 nm range spreading over all delays, that can be assigned to an unknown reaction intermediate. To gain more information about the species, we used a complex data deconvolution code SK‐Ana.^[^ ^38^ ^]^ Results of the analysis are visualized in Figure 2b,c. We can see that the dynamics of the new species does not correlate with the solvated electron decay and the species absorbs in a broad feature peaking at 520 nm. The species is formed upon the initial radiation pulse before the electron solvation occurs. Since the experiments are done in ethanol, with high probability the species originates from the interaction of quasi‐free electrons and the solute. In fact, the amount of this species depends on the initial concentration of the solute, supporting this conclusion.

The raw data from the measurement on VD2 line using pulse radiolysis with ns‐ms time scale, at wavelengths ranging from 350 to 570 nm, and streak camera for detection are in Figure 3. Based on them, a signal for a newly formed species absorbing around 350 nm can be observed. The transient species is present in the solution till the end of the measurement at 840 ns and its kinetics is correlated with the kinetics of solvated electrons (panel c). Therefore, this species is a product of the reaction of solvated electrons with RRx‐001 molecules. In the panel b of the Figure 3, the absorption spectrum of this intermediate is plotted as it was recorded at time of 75 ns after irradiation. Around this time, the absorption peak reaches its maximum in the whole time scale. The identity of newly formed transient species is discussed in more detail below.

**Figure 4:**
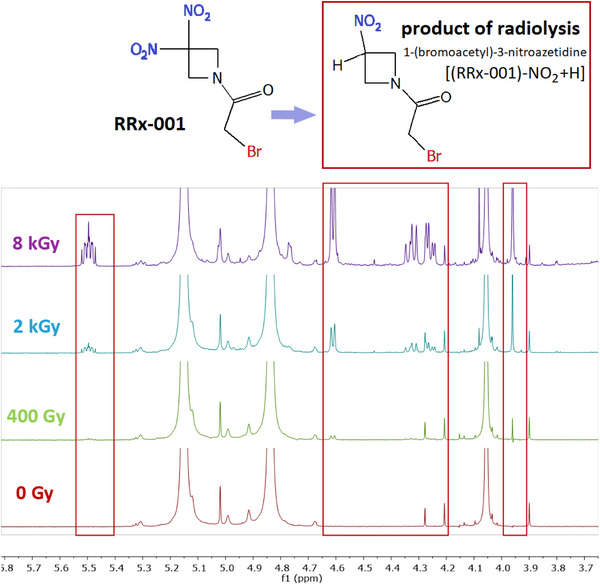
Structural patterns of molecules RRx‐001 on the left side and the resulting product of its radiolysis in ethanol solution—neutral molecule 1‐(bromoacetyl)‐3‐nitroazetidine—on the right side. The bottom panel shows 1H and 13C NMR spectra of 12 ml of RRx‐001 solution in ethanol after irradiation with accelerated electrons on microtron with three different radiation doses (400, 2000, and 8000 Gy). Radiolysis led to the loss of the nitro group and hydrogen/proton transfer generating 1‐(bromoacetyl)‐3‐nitroazetidine. Rectangles highlight the areas where the signal of the radiolysis product appears, particularly the methine group at 5.46‐5.53/71.7 ppm (δ_*H*_/δ_*C*_). Conversion of RRx‐001 to product was <1%, 4%, and 13% at 400, 2000, and 8000 Gy doses, respectively.

So far, we have discussed the experimental studies of LEE interactions with RRx‐001 in ethanol on a short time scale, when the intermediates are forming. To identify the stable products of radiolysis on the long timescales, we performed a radiolysis study upon irradiation at the Microtron facility of NPI in a range of delivered doses up to 8 kGy of 16.5 MeV electrons. Stable radiolysis products were identified by NMR spectroscopy. The spectra are shown in Figure 4. First, we observed that the RRx‐001 is highly stable upon irradiation, only at very high doses in kGy range a new radiolysis product of the molecule appear, which was identified as 1‐(bromoacetyl)‐3‐nitroazetidine, forming by substitution of the nitro group by hydrogen. The structural pattern of this radiolysis product is drawn in Figure 4. The evaluated conversion of RRx‐001 was <1, 4, and 13 molar% at 0.4, 2, and 8 kGy doses, respectively.

## 3 Discussion

**Figure 5:**
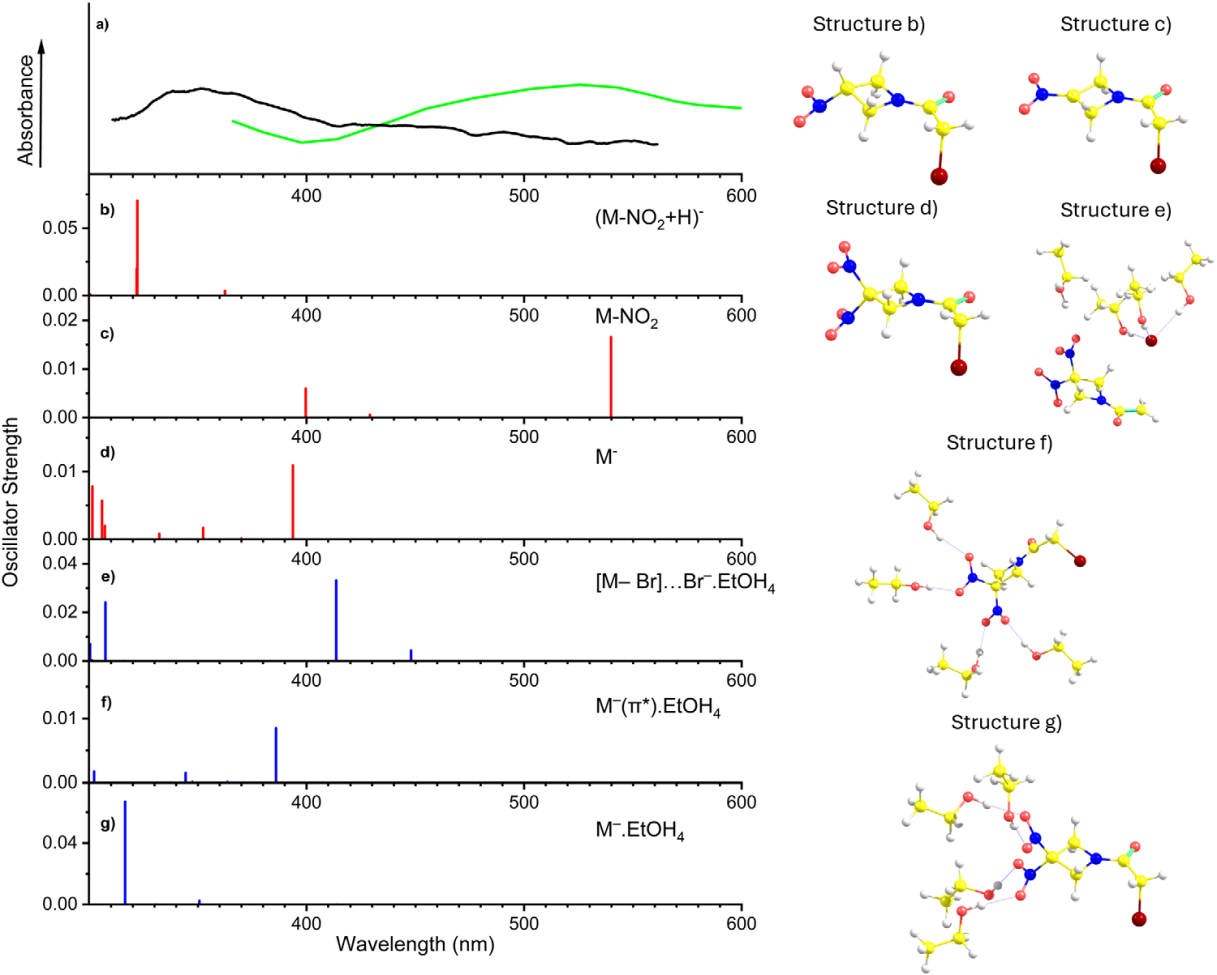
The comparison of absorption spectra obtained in pulse radiolysis experiments, panel a), with absorption spectra of possible intermediates based on computational modeling (TD‐CAM‐B3LYP/aug‐cc‐pVDZ), panels b–h). On the right side of the figure, we are showing the corresponding optimized structures, whose coordinates are in Supporting Information. The color scheme of the individual atoms is following: white = hydrogen, yellow = carbon, blue = nitrogen, red = oxygen, and brown = bromine. Panel a contains the absorption spectrum of a reaction intermediate forming upon interaction with solvated electrons, a black curve based on data from Figure 3 and of a reaction intermediate forming upon interaction with quasi‐free electrons, a green curve based on data from Figure 2. The absorption band of the intermediate whose kinetics correlate with solvated electrons overlaps well with the signal of [M‐NO_2_+H]^−^ forming after the loss of NO_2_ moiety, hydrogen transfer from solvent, and parent anion of RRx‐001. The absorption band of the intermediate whose kinetics does not correlate with solvated electrons overlaps only with the neutral [M‐NO_2_] signal. Panels e–g) show absorption spectra of parent anions in different states with four ethanol molecules. See Supporting Information for coordinates of the structures.

The identification of intermediates would not be conceivable without theoretical modeling. First, absorption spectra of the possible candidates for reaction products were computed through density functional method (DFT, see the Supporting Information for details) in implicit ethanol solvent. It is important to note that in the studied wavelength range, we are dealing with only tiny energy differences. For example, the energy difference for photons having wavelengths of 450 and 500 nm is less than 0.28 eV. The calculation errors for absorption spectra could be easily in this range, particularly considering a range of conformations accessible in the solvent. The spectra therefore provide only a first guess of the species for interpretation. The possible candidates were selected based on the described results of cluster and NMR experiments as well as the previous study of the isolated molecule.^[^ ^36^ ^]^ Figure 5 shows the calculated spectra of species that we could observe using the present experiments and are relevant for the discussion. Spectra for species whose absorption does not fit the experimentally accessible range are presented in the supporting material. The experimentally measured transient absorption spectra are shown in panel a to enable comparison.

In Figure 5, we can see that computed spectrum of two species overlaps with the absorption band at ∼350 nm corresponding to the signal of reaction intermediate forming upon interaction with solvated electrons. These are anion of RRx001 molecule upon NO_2_ substitution with hydrogen and parent anion.

The first species (Figure 5b) absorbs at the low wavelength edge of the observed band; however as mentioned, the calculated band position can be easily shifted with respect to reality. Additionally, this species is the anionic counterpart of the final radiolysis product observed on the long timescales, and therefore its observation cannot be excluded from the assignment. On the other hand, we have not observed this anionic product in cluster experiments. There are three possible explanations for that: i) the intermediate does not form, ii) the lifetime of the intermediate with respect to electron autodetachment is shorter than the detection time in our experiment (consider a maximum of absorbance at 75 ns and detection times in TOF experiments of tens of µs), iii) all reaction products, the dissolved anion, NO_2_ neutral, and EtO radical upon hydrogen abstraction stay within the cluster, which is detected as M(EtOH)^−^.

The second species is the parent anion (Figure 5d), whose formation is in agreement with our cluster experiments, where we demonstrated the stabilization of the parent anion in the solvent.

Since several initial states were found for the parent anion of RRx‐001 in our study of the isolated molecule,^[^ ^36^ ^]^ we decided to better explore the absorption spectrum of this anion. For that, the anion was modeled with explicit ethanol molecules and in different possible states. The resulting spectra are shown in Figure 5, panels e–g. The spectrum at panel f corresponds to the M…Br complex anion. Formation of such anion structures has been reported previously for several Br‐containing molecules, e.g., refs. [39, 40]. These complex anions can be important intermediate species in electron interactions, as they provide local minima that allow for both dissociation and anion stabilization dynamics. However, its absorption band lay exactly in the local minima of the experimentally detected absorption curves, making such an assignment improbable. Panels f,g correspond to the RRx‐001 parent anions in the lowest π* states. The spectrum on panel f corresponds to the electron delocalized over both NO_2_ groups, while the spectrum on the bottom g to the electron localized on a single NO_2_ group. We can see that these spectra fit the observed absorption band much better. This is the first indication that the interaction of RRx‐001 with secondary LEEs occurs exclusively via the π* resonances localized over the nitro group. The detected reaction products provide the second indication. We do not detect any dissociation products from breaking C─Br bonds. For example, the [M‐Br] radical upon dissociation of Br atom should be detectable using absorption spectroscopy at around 400 nm (see Supporting Information). In cluster experiments, the corresponding fragment anions could be also detected, e.g., in the form of solvated products, similar to our studies of thiophenols.^[^ ^40^ ^]^ Finally, NMR spectroscopy can easily detect final dissociation products. Based on this, we believe that the observed behavior is related to the inability of the LEEs to access the σ* state of RRx‐001. As we have shown in the study of isolated RRx‐001,^[^ ^36^ ^]^ the vertical attachment to this state occurs at energies around 0.5 eV, while π* states are energetically below the neutral. It seems that even the high electron affinity of the neutral RRx‐001 of 4 eV is not enough to rearrange the molecule in the solvent.

Considering the new species forming on a *ps* timescale, before the electrons are completely thermalized, its absorption, shown by the green curve in panel a of Figure 5, overlaps only with a single specie, which is parent after loss of the nitro group. This could be formed as a neutral product of electron interaction as well as in any other process during the radiolysis. The neutral products cannot be detected in the cluster experiments, that points to the limitations of electron attachment spectroscopy and the need for the development of new neutral detection techniques.^[^ ^41^, ^42^ ^]^ To link the observation of this intermediate product with the final product [M‐NO_2_+H], we can consider the following neutral reaction:

(1) $$\begin{array}{c} \left[M-{\mathrm{NO}}_{2}\right]+\mathrm{EtOH}⟶\left[M-{\mathrm{NO}}_{2}+H\right]+\mathrm{EtO}. \end{array}$$

This reaction is, however, endothermic by 0.39 and 0.36 eV as calculated in ethanol at B3LYP/aug‐cc‐pVDZ and ωB97XD/aug‐cc‐pVDZ levels, respectively. In the present environment full of electrons, anionic pathways also exist that are, on the other side, strongly exothermic.

(2) $$\left[M-{\mathrm{NO}}_{2}\right]+\mathrm{EtOH}+e_{\text{quasi-free}}^{-}⟶\;\left[M-\;{\mathrm{NO}}_{2}+H\right]+{\mathrm{EtO}}^{-}+4.0\mathrm{eV}$$

or

(3) $$\left[M-{\mathrm{NO}}_{2}\right]+\mathrm{EtOH}+e_{\text{quasi-free}}^{-}⟶\;{\left[M\;-{\mathrm{NO}}_{2}+H\right]}^{-}+\mathrm{EtO}+2.9\mathrm{eV}$$

The energies were calculated at B3LYP/aug‐cc‐pVDZ level of theory. Equation (3) will require an electron autodetachment step to explain the final NMR product. Electrons can therefore play a catalytic role^[^ ^43^ ^]^ in the hydrogenation of the [M‐NO_2_] toward the final product detected by NMR. The main energy supply for this process then originates in the high electron affinity of the EtO and [M‐NO_2_+H] anions of 4.4 and 3.3 eV, respectively, as calculated in ethanol at the B3LYP/aug‐cc‐pVDZ level (ωB97XD/aug‐cc‐pVDZ values are 4.4 and 3.1 eV).

**Figure 6:**
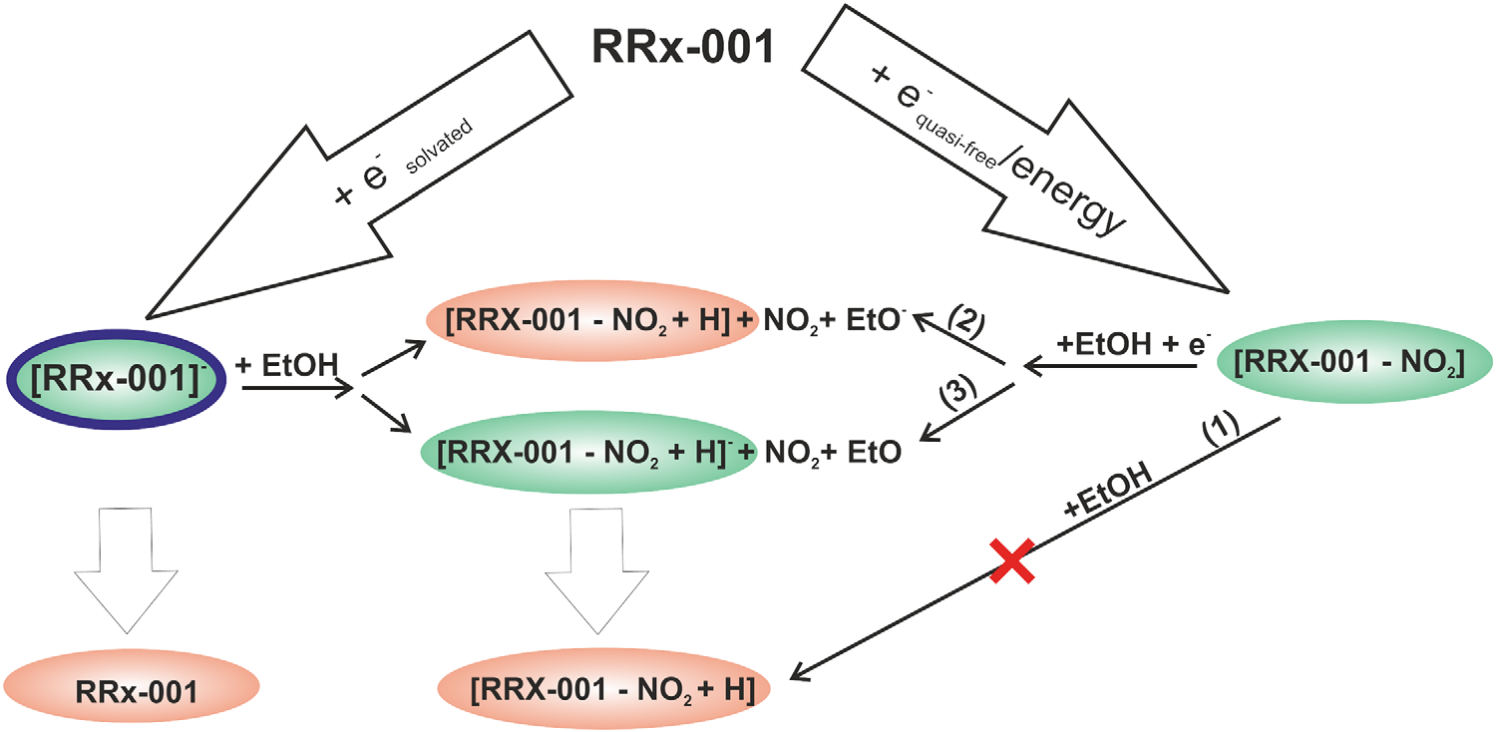
Suggested low‐energy electron pathways of the RRx‐001 radiolysis. In red are the final products detected by NMR spectroscopy. The intermediate RRx‐001^−^ product identified in cluster experiments is in blue. In green are intermediate products that could be assigned by combining time‐resolved absorption spectroscopy with computational modeling. RRx‐001 strongly scavenges both solvated and quasi‐free electrons. Interaction with quasi‐free electrons, or possibly other energetic processes during the initial radiolysis pulse, results in the formation of [M‐NO_2_] neutral. This neutral product can be transformed into the final 1‐(bromoacetyl)‐3‐nitroazetidine product; however, only in the presence of the low‐energy electrons catalyzing the ethanol dehydrogenation reaction. The numbers (1), (2), (3) above the corresponding reaction path arrows indicate the reactions described in the text. Solvated electrons interact with the molecule by the formation of the parent anion in a reversible process. A small part of the parent anions can undergo a dissociation to form the final reaction product 1‐(bromoacetyl)‐3‐nitroazetidine in an exothermic reaction. Possibly, also the 1‐(bromoacetyl)‐3‐nitroazetidine anion can contribute to the observed absorption, which correlates with solvated electron kinetics; however, the combined experimental and computational errors do not allow for unambiguous assignment of this intermediate.

The discussion therefore leads to the reaction mechanism that is sketched in Figure 6. RRx‐001 is a strong scavenger of electrons, what may be due to its huge electron affinity in the ethanol of 4 eV. NMR spectroscopy of the solution irradiated with extreme doses indicates that despite the high reactivity with LEEs, the molecule remains very stable, and only a small portion of the molecule is converted to 1‐(bromoacetyl)‐3‐nitroazetidine neutral product. Final products of radiolysis as well as RRx‐001 parent anion intermediate were experimentally detected and can be therefore assigned unambiguously, they are highlighted red in Figure 6. The species highlighted in green were assigned by combining transient absorption spectroscopy with ab initio calculations, and their assignment is tentative. Still, they allow us to postulate the reaction mechanism based on hydrogen transfer from ethanol shown in the central part of Figure 6 corresponding to Equations (2) and (3). We can also see that all the pathways are solely on NO_2_ group, while the Br atom and its corresponding sigma orbitals seem to be inactive in the process.

## 4 Conclusion

The present study of RRx‐001 radiation chemistry profits from a highly complex and unique combination of experimental techniques and computational modeling. We experimentally demonstrate the high reactivity of RRx‐001 in solution with secondary LEE forming upon ionizing radiation event in both quasi‐free and solvated electron form. The C37 constant, defined as a molar concentration of the substance needed to scavenge 63% of quasi‐free electrons, was measured to be 0.2 M (room temperature, atmospheric pressure). For the reaction with solvated electrons, the evaluated rate constant is equal to 9.4×10^9^ dm^3^mol^−1^s^−1^, which practically means diffusion‐controlled reaction. Despite this high reactivity, RRx‐001 remains very stable in the solvent up to kGy radiation doses, which can be explained by the creation of transient negative ions of RRx‐001 in solution. These were unambiguously identified in both, gas phase and bulk experiments on different timescales. We can see that the gas phase studies with ballistic electrons can be correlated with pulse radiolysis studies when all the experimental conditions are under control. However, we can also see that not all the states accessible by ballistic electrons can be accessed in solution. A combination of transient absorption spectroscopy in solution and NMR spectroscopy of final radiolysis products then identify the important neutral dissociation pathways as well.

Most intriguing is then the fact that despite the high electron affinity of Br atom and gas phase exothermicity of Br anion release from RRx‐001, in solution the Br localized anions seem to be inactive. We assign this to the inaccessibility of high‐lying σ* virtual states known for isolated molecules^[^ ^36^ ^]^ by electrons in solution. This can contribute to the long‐standing discussion about the importance of different electron attachment states in solution and their relevance for radiation‐induced biomolecular damage. The discussion was particularly intense in two fields, i) DNA and RNA damage by low‐energy electrons^[^ ^44^, ^45^, ^46^, ^47^, ^48^, ^49^, ^50^ ^]^ and ii) radosensitizers^[^ ^37^, ^51^, ^52^ ^]^ where the RRx‐001 belongs to the second group. However, solvated anions are essential in many other fields such as atmospheric chemistry, plasmonics, or catalysis.^[^ ^53^ ^]^ Based on the present observation, the accessibility of the initial virtual state in solution seems more important than reaction enthalpy. Precise prediction of vertical attachment energies in solutions (e.g., ref. [54]) will be crucial for modeling the secondary electron contributions to the complex radiation damage.

## Conflict of Interests

The authors declare no conflict of interest.

## Supporting information

Supporting Information

## Data Availability

The data that support the findings of this study are available in the supplementary material of this article.
