# Supplementary material for: Nitro‐Group π System Drives the Interaction of RRx‐001 with Electrons in Solution
Source: Chemistry. 2025 May 9;31(31):e202500859. doi: 10.1002/chem.202500859 (PMC12133630; doi:10.1002/chem.202500859)
Supplement: Supplementary file 1 — Supporting Information [file CHEM-31-e202500859-s001.pdf]

Supporting information to: Nitro-group π system drives the interaction of RRx-001 with solvated electrons in solution.

Barbora Sedmidubská,[a,b,c] Sergey Denisov,[b] Mehran Mostafavi,*[b] Stephan Denifl,[d,e] Farhad Izadi,[d,f] Milan Ončák,*[d] Thomas F.M. Luxford,[a] David Chvátil,[g] Jiří Pinkas,[a] Jaroslav Kočišek *[a]

Contents

1. Methods 2
1.1. Electron attachment spectroscopy of clusters 2
1.2. Pulse radiolysis 2
1.3. Irradiation on microtron + NMR spectroscopy 3
1.4. Ab initio calculations 3

2. Kinetics of RRx-001 interaction with secondary low-energy electrons 4

3. CLUB Measurements 6

4. Description of NMR spectra of final radiolysis product 1-(bromoacetyl)-3-nitroazetidine; C5H7BrN2O3; M=223 9

5. Calculated absorption spectra 10

6. Cartesian coordinated of optimized structures 11

7. References 17

[a] Dr. B. Sedmidubská, Dr. T. F. M. Luxford, Dr. J. Pinkas, Dr. J. Kočišek*
J. Heyrovský Institute of Physical Chemistry of the CAS, Dolejškova 3, 182223 Prague, Czech Republic
E-mail: kocisek@jh-inst.cas.cz

[b] Dr. B. Sedmidubská, Dr. S. Denisov, Prof. M. Mostafavi*
Institut de Chimie Physique, Université Paris-Saclay, CNRS, 91405 Orsay, France
E-mail: mehran.mostafavi@universite-paris-saclay.fr

[c] Dr. B. Sedmidubská
Department of Nuclear Chemistry, Faculty of Nuclear Sciences and Physical Engineering, Czech Technical University in Prague, Břehová 7, 11519 Prague, Czech Republic

[d] Prof. S. Denifl, Dr. F. Izadi, Prof. M. Ončák*
Universität Innsbruck, Institut für Ionenphysik und Angewandte Physik, Technikerstraße 25, 6020 Innsbruck, Austria
E-mail: Milan.Oncak@uibk.ac.at

[e] Prof. S. Denifl
Center for Molecular Biosciences Innsbruck, Universität Innsbruck, Technikerstraße 25, A-6020 Innsbruck, Austria

[f] Dr. F. Izadi
Department of Chemistry and Biochemistry, Texas State University, San Marcos, TX 78666, USA

[g] Dr. D. Chvátil
Department of Accelerators, Nuclear Physics Institute of the CAS, 25068 Rez, Czech Republic

1. Methods

The sample of RRx was purchased from AmBeed with a declared purity of 95.00% and the sample was used in all the present experiments. The primary source of information for the present contribution is provided by the pulsed radiolysis combined with absorption spectroscopy to identify the reactivity of the pre-solvated and solvated secondary low-energy electrons towards the molecule. Identifying the initial steps of such electron reactions in the solvent is extremely complicated due to the short lifetime and short mean free path of LEEs in solvent reducing the probability of the interaction with the molecule of interest. Even the state-of-the-art pulsed radiolysis experiment described requires high dose rates of radiation to only indirectly identify such interactions. Therefore, we employed experiments colliding low-energy electrons with microsolvated RRx-001 molecule isolated in a vacuum, enabling the identification of transient anions formed in the initial electron interaction step. Finally, the long-lived radiolysis products of RRx radiolysis were identified combining the radiolysis with nuclear magnetic resonance spectroscopy. The experimental results are then interpreted with the support of the state of the art computational modeling.

1.1. Electron attachment spectroscopy of clusters

The cluster experiments were performed at the cluster beam apparatus (CLUB). It is a universal device to investigate molecules in the molecular beam in their isolated form or in clusters, exploring the environmental effects on the reaction. The device contains several vacuum chambers and tools for studying the dynamics of molecules and their clusters.[1,2] In this work, we used only the molecular beam source and reflectron time-of-flight mass spectrometer (rTOF) in a configuration identical to our previous studies.[3] The sample in the form of white crystal powder was inserted into a metal borosilicate glass dish inside a stainless steel vessel in the inlet system and sublimed at a temperature of approximately 110°C. Then vapors of RRx-001 were co-expanded in the flow of Ne as a carrier gas through the conical 90 μm nozzle into the vacuum. To gradually increase the level of micro-solvation by ethanol and water(see SI), the carrier Ne gas was transferred through a tube made of the Nafion membrane, submerged in the solvent. Molecules of solvent from the solution penetrated via the membrane and were carried by Ne gas molecules to the nozzle mixing with RRx-001 vapors. The adiabatic expansion into vacuum cools the mixture in a way that small aggregates - clusters are formed. The size of the clusters was, to some extent, modified by the temperature of the nozzle and pressure of the carrier gas, which are stated together with the respective data sets. The molecular beam was then crossed by an electron beam in a reaction chamber under the right angle of 90°. Electrons were emitted from a hot wire and accelerated to the energies from 0 to 12 eV. The energy scale was calibrated on a characteristic 4.3eV resonance of O^- arising from dissociative electron attachment to CO_2.[4] The ions resulting from the interaction of the molecular beam with the electrons were extracted to the rTOF, where they were analyzed according to their time of flight.

1.2. Pulse radiolysis

The radiolysis of RRx molecule in ethanol at various concentrations was studied on the ELYSE platform (electron accelerator)[5], which enables to performance of both pulse radiolysis in a wide range of times: from picoseconds to 11 ns and microsecond ranges. In two experimental setups for sub-microseconds and microseconds, the core remains the same. The C-fiber 100 fs Er laser generates a fundamental beam at 780 nm, which is tripled ca. 260 nm for electron photo extraction from the CS_2Te photocathode, that are accelerated up to 7.8 MeV to form 7 ps pulses with a frequency of 5 Hz. The pulses of high energy electrons are used for irradiation of sample solution in the quartz cell on the direct line VD (ps pulse radiolysis) or on the bend line VD2 (ns-ms pulse radiolysis time scale experiments). Experiments were carried out in a static cell (2.5 ml of solution) or in a cell with constant circulation (25 ml of solution) to prevent overdosing of irradiated volume or provide a homogeneous concentration in case of approaching the solubility limit. Cells have an optical path of 0.5 cm, and an optical window has a thickness of 200 μm. Circulation in the cell is achieved using a closed tube loop with a solution. The circulation can reach a rate of around 60 cm^3/min. For more details, see.[5,6]

Picosecond pump-probe experiments

Picosecond pulse radiolysis was carried out on the direct line VD with the transient absorption pump-probe set-up. In this setup, the part of the light from the Er laser used for creating photoelectron pulses is deflected towards a CaF_2 crystal creating a supercontinuum light pulse with wavelength in 360 nm to 700 nm range. This pulse can be mechanically delayed from the constant arrival of an electron pulse to up to 11.5 ns. The pulse is further split to pass via a reference path or the sample cell and the differences due to the sample absorption are analyzed using spectrometertype and detected using CCD camera. A picosecond pump probe line VD has an time window of 11.5 ns, a spectral window from 360 nm to 700 nm and an excellent resolution of 150 fs.[5,6]

Nanosecond to millisecond time scale experiments

Nanosecond to millisecond time scale experiments are carried out on the bend line (VD2) and besides the core described above, it contains the detection part which includes a flash xenon lamp as a source of light for analysis. A light passing through the irradiated solution goes to the highly dynamic streak-camera (Hamamatsu C7700-01) in connection with a spectrograph for detection (AndorKymera 328i). The xenon lamp was triggered with respect to the electron pulse with possible delays in 1 ns to 1ms range. The spectral window ranges between 350 nm and 600 nm and a time resolution is 50 ps (for the 1 ns time window).[5,6]

1.3. Irradiation on microtron + NMR spectroscopy

Final products were studied using NMR spectroscopy using Bruker AVANCE NEO 500MHz spectrometer applied on ethanol solution of RRx irradiated by different doses of high energy 16.5 MeV electrons from Microton MT25.

Microtron MT25 is a cycle accelerator of electrons containing a Kapitza's resonator. Electrons are accelerated by a high-frequency electric field with constant amplitude and frequency in a homogeneous magnetic field. In a vacuum chamber, electrons move along circular paths with a common tangent point, where a cavity resonator is located, and are extracted from a particular circular trajectory defined by electron energy. The electron beam is pulsed with a pulse length of 3.5 µs and a repetition rate of 423 Hz. The mean current was about 20 µA. In this work, the electron energy of 16.5 MeV was applied. The sample solutions were irradiated in 15 ml centrifuge tubes made of polypropylene. After irradiation, solutions were poured into spare tubes to prevent possible interaction between the studied solution and the inner side of the irradiated plastic tube. This measurement was one of the first and we did not displace air with Ar gas. However, our later experiments showed that displacement (reducing the electron scavenging capacity of diluted oxygen) has only a small effect on overall dose dependence of the measurement, however, it did not affect the qualitative determination of the radiolysis product, which we primarily aimed for.

1.4. Ab initio calculations

Selected molecules and ions were optimized at the B3LYP/aug-cc-pVDZ level in implicit ethanol solvent described within the polarizable continuum model (PCM). For selected molecules, explicit ethanol molecules were added. In the resulting structures, electronic transitions were calculated at the TD-CAM-B3LYP/aug-cc-pVDZ level. Wave function stabilisation was performed prior to each calculation. The Gaussian software was employed. [7]

2. Kinetics of RRx-001 interaction with secondary low-energy electrons

Picosecond pulse radiolysis measurements of the kinetics of solvated electrons at 600 nm were performed for ethanol solutions of RRx-001 molecule with concentrations (0, 10, 25, 50, 80 mM) on the direct line the ELYSE platform described in the Methods section. The maximum concentration of 80 mM is still low enough to exclude direct radiolysis of the solute. The graph plotted on the left side of Fig. S1 shows the evolution of the absorption band at 600 nm of the solvated electron on a short time scale up to 30 ps. The time evolution starts with a zero signal in the region before the electron pulse of accelerated electrons. Then, a steep signal increase occurs reaching its maximum value within the tens of picoseconds, during and right after the electron pulse. In fact, as the dose per pulse is constant for the different experiments reported in Fig. S1, if there is no reaction between the precursors of the solvated electron and the solute we had to obtain the same absorbance after the pulse at 600 nm. However, the maximum of absorbance at 600 nm, corresponding to the amount of the solvated electrons per pulse, decreases with an increase in the concentration of the RRx molecule. Since the initial amount of the secondary electrons produced by the pulse is in average constant, the decrease indicates the reactivity of the electrons with the RRx-001 molecule before the solvation process. Therefore, we can confirm that the precursors of the solvated electron, i.e. quasi-free electrons, are scavenged very fast by the solute. The scavenging efficiency is indirectly reported in radiation chemistry by the value of C37. This value represents the concentration of the scavenging molecule in the solution at which the initial amount of solvated electrons in the solution is 37% ($1/e$), i.e. 63% [1-($1/e$)] of all very low-energy electrons have already been scavenged in the form of quasi-free electrons by a studied scavenger. The procedure to obtain this value is based on the relation of radiation-chemical yield G of solvated electrons, the absorbance A, the concentration c of the studied scavenger, and the *C37* value for select scavenger in a specific solvent. A brief explanation of this value and its calculation based on radiation-chemical yields of solvated electrons can be found e.g. in the works of K. Iwamatsu and M. Mostafavi [8,9]. In our case, the procedure to obtain the C37 value was as follows.

The relationship between the radiation-chemical yield of solvated electrons and the concentration of scavenger in solution and C37 value is well defined in the equation 2.1.,

$$\frac{G(c)}{G(c=0)} = e^{-\frac{c}{C37}}, \tag{2. 1}$$

where G(c) [mol/J] is a radiation-chemical yield of solvated electrons formed in solution under a certain applied concentration of a studied scavenger, G(c=0) [mol/J] is the radiation-yield of solvated electrons formed in solution without a presence of a studied scavenger, c [mol/l] is the applied concentration of a studied scavenger, and C37 [mol/l] is the specific concentration of the studied scavenger when the concentration of formed solvated electrons in solution reaches 37%.

Simultaneously, there is a correlation between radiation-chemical yield of solvated electrons G and absorption A of solvated electrons in solution shown via the equation 2.2.

$$G(t) = \frac{A_{e^-_{aq}}(\lambda t)}{\epsilon_\lambda \cdot d \cdot F \cdot D}, \tag{2. 2}$$

Where G(t) [mol/J] is a radiation-chemical yield of solvated electrons in specific time, $A^-_{e\ aq}$ is absorption of solvated electrons at the certain wavelength, ϵ_λ is molar attenuation coefficient for specific wavelength, d is a optical path-length and F is a dose factor, more explained in previous articles [9], D is a given dose.

Based on these two equations and since the parameters for measurements in the denominator of the equation 2.2 remain the same in case of measurements in the ethanol solvent with or without scavenging molecule, then the C37 can be reached from the final relationship represents by an equation 2.3 and linear fit of plotted data on the right graph in the Fig.S1:

$$ln\frac{A(c)}{A(c=0)} = -\frac{c}{C37} \tag{2. 3}$$

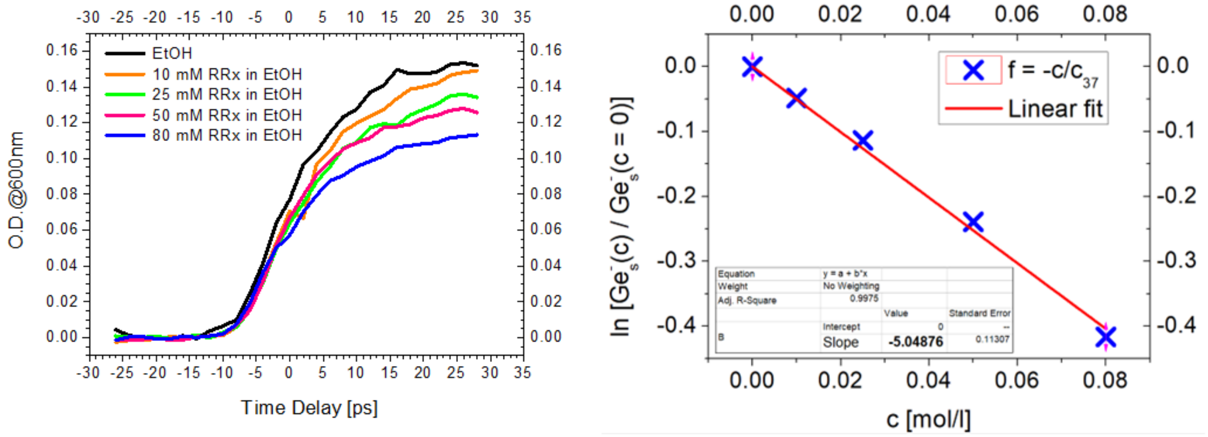

Figure S1. The figure containing kinetics of solvated electrons in graph on the left side up to 35 ps after absorption of a 5 ps electron pulse by a sample of ethanol (black curve), and RRx-001 in ethanol solution with various concentrations (other curves). The graph refers to scavenging of presolvated electrons by RRx via the drops of curves with increasing concentrations of RRx-001 in zero time. Right-side graph shows the values of the $K_{observed}$ versus concentration. The slope of the linear fit to the data represents the value of the absolute rate constant of the reaction between the solvated electrons and RRX in solution.

Kinetics of the reaction with solvated electrons can be extracted from the data depicted in the left graph of Fig.S2 Here the same concentration dependence of the absorbance signal discussed in the previous paragraph was measured on a longer time scale up to 10 ns using the bend line VD2 of the platform ELYSE. The graph displays the decay of solvated electrons at 600 nm versus time. With increasing concentration of the RRx-001 solute, the decay is faster. The most powerful difference can be seen when selecting the kinetics of the solvated electron in neat ethanol solution and ethanol solution of the studied molecule with 80 mM concentration. Here, we clearly show through the strong correlation between the RRx-001 concentration and the changes in absorbance of solvated electrons that they strongly interact with RRx-001. To quantify the reaction rate of the solvated electron scavenging, we show in the graph on the right side the value of the $K_{observed}$ versus concentration. The data points are linearly plotted and the slope value represents the value of the absolute rate constant of the reaction between solvated electron and RRX in solution.

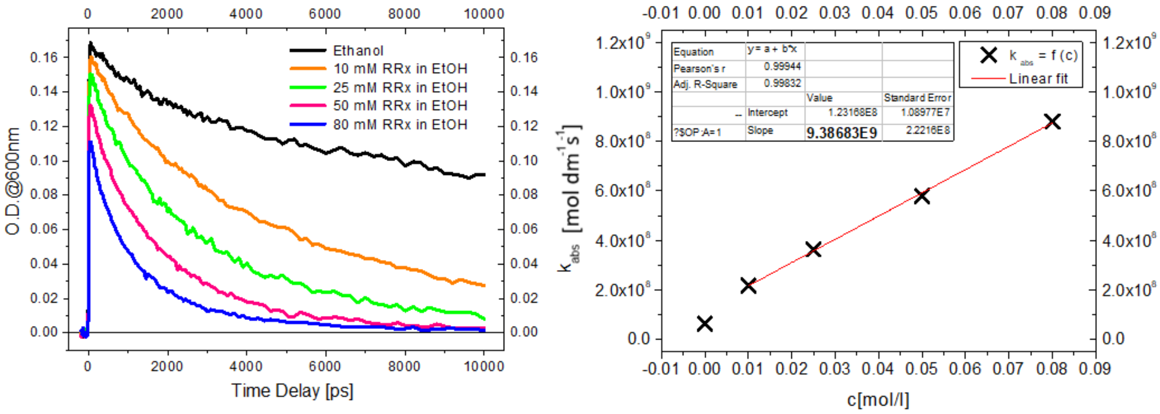

Figure S2. Figure shows graphs characterizing the interaction of RRx with solvated electrons. Left side graph contains the kinetics of solvated electrons up to 10 ns after absorption of a 5 ps electron pulse by a sample of ethanol (black curve), and RRx-001 in ethanol solutions with various concentrations (other curves). The graph on the right side plots five values of absolute rate constant in dependence on their respective concentration of RRx in ethanol solution. The data are plotted with linear fit when the slope value represents normalized rate constant which characterizes rate of scavenging of solvated electrons by RRx in ethanol solution.

3. CLUB Measurements

The experiments were performed at sample temperatures right above the observation threshold for the molecular anion to ensure that there is no thermal decomposition of the sample or formation of dimer cations of RRx-001. Therefore, the measured intensities are extremely low. This is visible on the product intensity ratios for Br isotopes that do not fit well, since the background random variations influence the signal up to the 20%, similarly the background signal of NO_2 further complicated the measurements. In the main text we are showing background subtracted values of integrated intensities, raw data are shown in Fig. S3. Raw data for expansions in water are then shown in Fig.S4

The NO_2^- anion and parent anion of ethanol $EtOH^-$ have similar masses [m/z (NO_2^-) = 45.9929, m/z ($EtOH^-$) = 46.04187], which cannot be separated using our mass spectrometer. However, we do not expect significant contributions $EtOH^-$ at m/z 46, due to the extremely low probability of electron attachment at present low energies of electrons [10,11] and the fact that the primary electron attachment product of ethanol is the m/z 45 anion upon the DEA via hydrogen release, which is observed even in bulk solutions. [12]

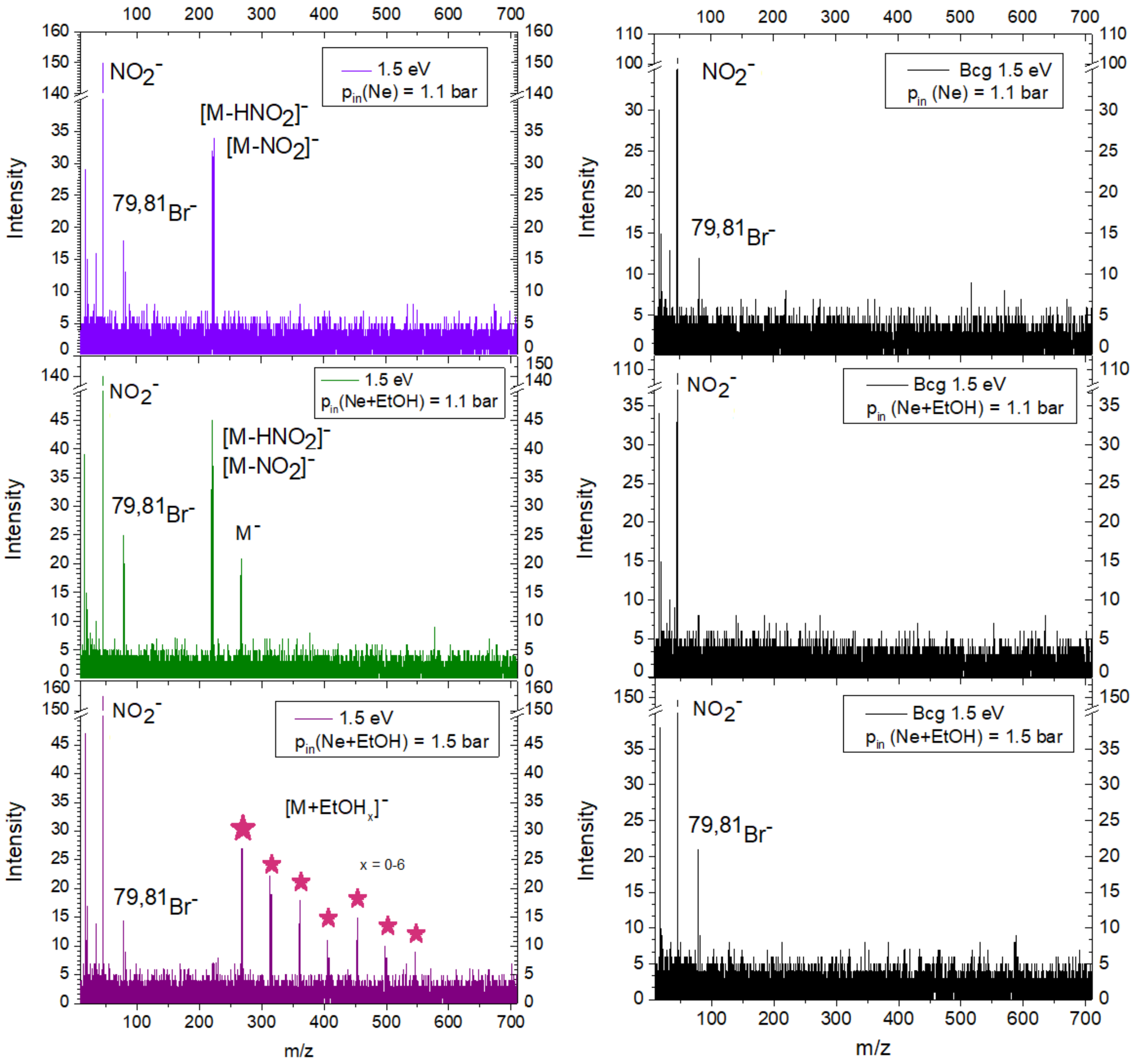

Figure S3. Mass spectra of negative ion fragments upon attachment of ballistic electrons to neutral mixed clusters of RRx-001 with ethanol (RRx-001)$EtOH_k$ in molecular beam experiments. The pressure of neon expansion gas is used to control the amount of ethanol k in the precursor cluster from zero ethanol solvation a) through medium b) to high c). We are reporting raw data plus corresponding background spectrum on the right panel.

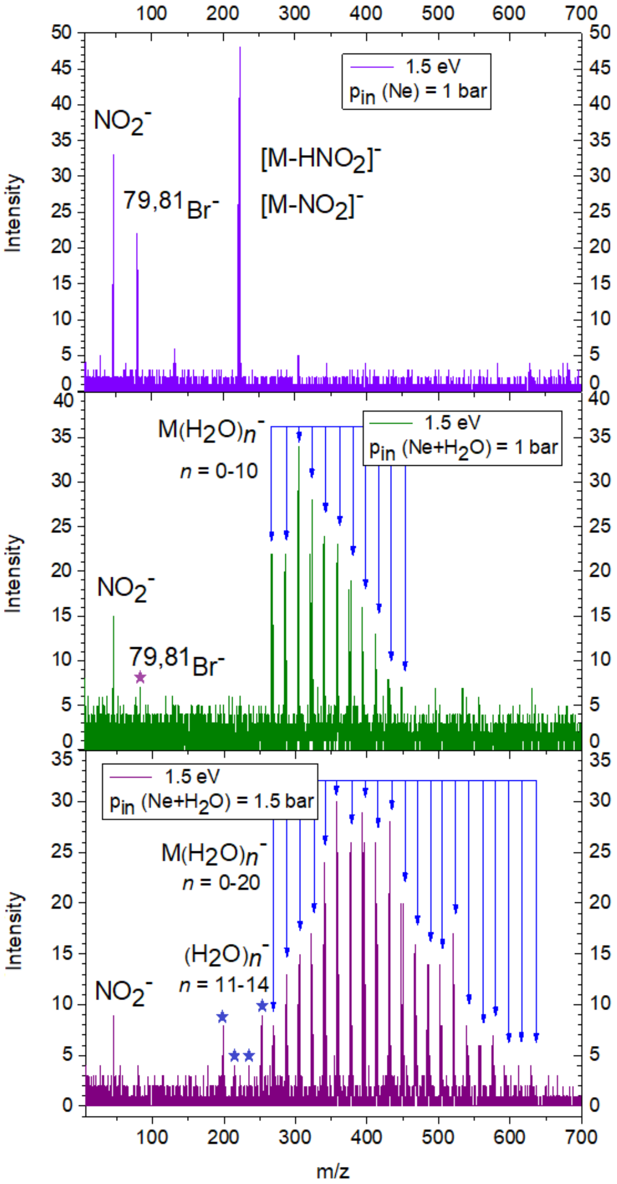

Figure S4. Mass spectra of negative ion fragments upon attachment of ballistic electrons to neutral mixed clusters of RRx-001 with water (RRx-001)H_2O_k in molecular beam experiments. The pressure of neon expansion gas is used to control the amount of water k in the precursor cluster from zero hydration a) through medium b) to high c). The expansion conditions were the same as for ethanol in Fig.S3.

Figure S5 shows energy-dependent ion yields in the present experiments for M^- and NO_2^- anions. The low energy signal in the present experiments spreads over a wide area from 0 eV to 4 eV. In contrast to our previous experiments, e.g. [13], in the present experiment the the parent and nitro anion signals cannot be separated based on the energy-dependent anion yields. Based on our experience this is caused by the settings of the electron gun to provide high currents that allow us to detect the present low-intensity signals. At these high currents, the electron energy distribution function of the spectrometer significantly deteriorates, particularly at low electron energies, due to the coulomb repulsion between electrons.

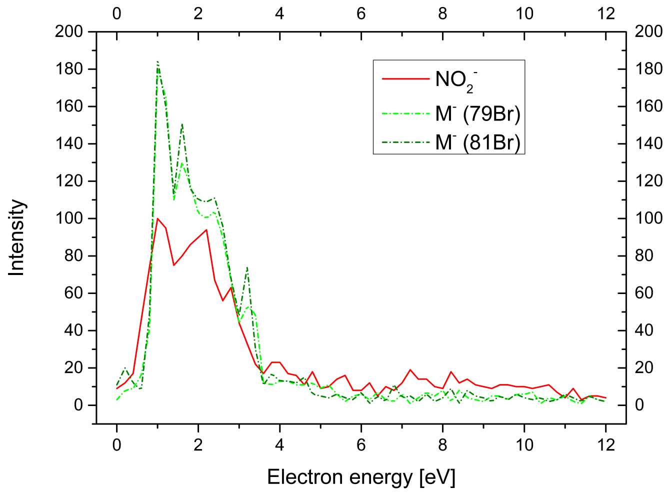

Figure S5. Electron-energy dependence spectra for the formation of the parent anion M^- and $NO_2{}^-$.

4. Description of NMR spectra of final radiolysis product 1-(bromoacetyl)-3-nitroazetidine; C5H7BrN2O3; M=223

^{1}H (dmso-d_6): 3.96 (s, 2H, CH_2Br); 4.23−4.36 (m, 2H, CH_2); 4.61 (d, $^3J_{HH}$ = 6.1 Hz, 2H, CH_2); 5.46−5.53 (m, 1H, CH). ^{13}C^{1}H (dmso-d_6): 26.0 (CH$_2$Br); 53.3, 55.3 (CH$_2$); 71.7 (CH); 166.1 (CO).
Note: RRx1-001 as well as 1-(bromoacetyl)-3-nitroazetidine product gradually decompose in dmso-d_6.
^{1}H (CDCl$_3$): 3.69 (s, 2H, CH$_2$Br); 4.51 (d, $^3J_{HH}$ = 5.9 Hz, 2H, CH_2); 4.68−4.79 (m, 2H, CH_2); 5.20−5.27 (m, 1H, CH). ^{13}C^{1}H (CDCl$_3$): 23.8 (CH$_2$Br); 53.9, 55.8 (CH$_2$); 71.2 (CH); 166.3 (CO). ^{15}N (CDCl$_3$, gHMBC): 97 (NCO); 382 (NO$_2$).

5. Calculated absorption spectra

Fig S6 shows the spectra of species that we cannot detect, their absorption does not fit the experimentally accessible range of wavelengths.

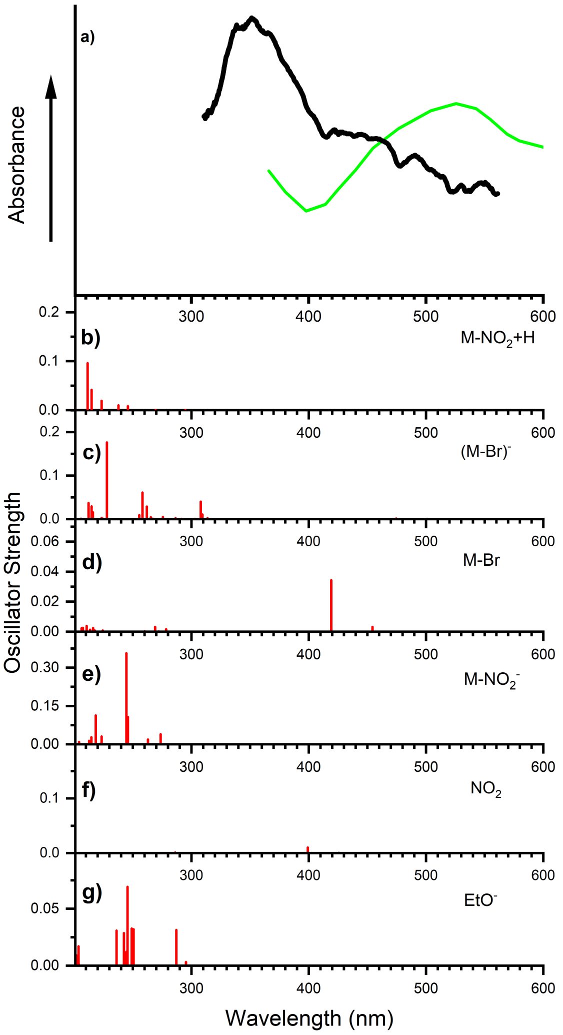

Figure S6. The comparison of absorption spectra obtained in pulsed radiolysis experiments panel a) with calculated absorption spectra of possible intermediates that cannot be detected in present experiments b) to g). Panel a) contains the absortion spectrum of a reaction intermediate forming upon interaction with solvated electrons, black curve and of reaction intermediate forming upon interaction with pre-solvated electrons, green curve. Absorption bands of these intermediates lay mainly below the detection threshold of the present experiment.

6. Cartesian coordinated of optimized structures

Cartesian coordinates (in Angstrom) and electronic energy including zero-point energy correction as optimized at the B3LYP/aug-cc-pVDZ level

$EtOH^-$
E = -155.029443
H -1.514415 -0.618239 0.771945
O -1.235130 -0.281142 -0.134056
C -0.072856 0.547612 0.028785
H -0.093040 1.283905 -0.787367
H -0.149736 1.102840 0.981696
C 1.231012 -0.245468 -0.005276
H 2.094171 0.437341 0.068783
H 1.275156 -0.952429 0.836385
H 1.319972 -0.817141 -0.940049

$[M\text{-}NO_2+H]^-$
E = -3104.042828
C 1.960951 0.605077 0.871417
Br 2.612145 -0.872020 -0.289527
C 0.868956 1.379281 0.153343
O 1.138597 2.368493 -0.546067
N -0.382295 0.939216 0.341291
C -1.647258 1.409615 -0.246344
C -2.373997 0.290526 0.545497
C -0.992327 -0.215284 1.035747
N -3.131592 -0.663214 -0.266572
O -4.047073 -0.120524 -1.034638
H -3.022148 0.664403 1.352636
O -3.343604 -1.823054 0.310739
H -1.902180 2.441196 0.023872
H -1.691440 1.284668 -1.334967
H -0.840146 -0.214493 2.121565
H -0.705254 -1.183967 0.610829
H 1.628958 0.130784 1.797662
H 2.823017 1.251498 1.040577

$[M\text{-}NO_2]$
E = -3103.277725
C -1.584621 1.332728 -0.327416
N -0.374006 0.862014 0.381285
C -0.948035 -0.458438 0.752061
C -2.184089 0.023846 0.067878
H -2.055471 2.227458 0.106721
H -1.444614 1.483674 -1.409376
N -3.436743 -0.529393 -0.103494
O -3.638754 -1.669089 0.366509
O -4.289759 0.142803 -0.720345
H -1.044419 -0.634003 1.833983
H -0.457926 -1.323309 0.278662
C 0.884534 1.349604 0.296610
O 1.133550 2.419973 -0.260978
C 1.966271 0.519255 0.961948
H 2.814155 1.157070 1.213835
H 1.616555 -0.041436 1.832056
Br 2.651494 -0.827122 -0.325383

M^-
E = -3308.590422
C 1.767857 0.033998 0.003691
N 2.251501 -1.156126 0.751571
N 2.941802 0.494505 -0.784155
O 3.172733 -0.103388 -1.871579
O 3.788745 1.213252 -0.185288
O 2.272001 -2.254530 0.131384

O 2.884669 -0.942456 1.821327
C 0.469589 -0.170816 -0.802162
C 1.069145 1.115573 0.852230
H 1.500660 2.116773 0.754755
H 0.948093 0.849853 1.907558
N -0.132369 0.889270 0.033725
H 0.559941 0.082153 -1.863244
H 0.019249 -1.160242 -0.674196
C -1.367537 1.398005 0.176413
O -1.640555 2.251369 1.030279
C -2.417771 0.878610 -0.789133
H -2.015647 0.592204 -1.763730
Br -3.261932 -0.758042 -0.043875
H -3.219665 1.610996 -0.888721

[M-Br]...$Br^{-}.Et_4$
E = -3928.557940
C -2.500430 -3.873048 0.055642
C -3.293761 -2.928073 0.813974
N -3.384891 -1.656587 0.314319
C -2.583314 -0.893452 -0.658854
C -3.209684 0.388292 -0.060883
N -2.234903 1.428684 0.451879
O -2.459769 1.930600 1.538728
O -3.901903 -3.230400 1.859454
C -3.925284 -0.483516 1.002288
N -4.169084 1.125034 -0.988988
O -4.942027 0.412850 -1.608403
O -4.119467 2.343573 -1.028278
O -1.293540 1.692583 -0.286795
Br 1.171035 -1.973754 -0.221849
H -2.421341 -4.890043 0.430807
H -1.981940 -3.598040 -0.860116
H -1.497119 -0.956205 -0.508542
H -2.843323 -1.068878 -1.707507
H -3.533645 -0.328056 2.014284
H -5.017853 -0.404729 1.001377
H 3.515303 -2.269440 -0.766080
O 4.457655 -2.393313 -0.988788
C 5.119959 -1.127834 -0.880221
C 6.585870 -1.314375 -1.231927
H 4.654737 -0.396790 -1.563274
H 5.021373 -0.735544 0.146382
H 7.118736 -0.356000 -1.156917
H 7.062710 -2.029458 -0.546967
H 6.695015 -1.691047 -2.258659
H 1.574817 -1.255285 2.056782
O 1.737900 -0.986254 2.980786
C 2.749949 0.027470 2.987138
C 3.029634 0.426508 4.425931
H 3.670154 -0.352010 2.511413
H 2.412049 0.904023 2.408446
H 3.803631 1.206090 4.459356
H 2.122014 0.819651 4.905070
H 3.383294 -0.436304 5.007350
H 0.901652 -0.666044 -2.245232
O 0.767739 -0.158931 -3.068210
C 1.546812 1.041445 -2.994410
C 1.313492 1.849609 -4.259271
H 1.255269 1.628640 -2.107997
H 2.616924 0.792629 -2.893741
H 1.902057 2.777201 -4.230231
H 1.613891 1.277358 -5.148110
H 0.252345 2.116669 -4.360348

H -0.056009 3.332811 0.078819
O 0.512175 4.116765 0.096157
C 1.261080 4.113076 1.321171
C 2.172012 5.327767 1.331182
H 0.573988 4.143777 2.182965
H 1.856102 3.187756 1.396161
H 2.759951 5.351104 2.259207
H 2.867802 5.299750 0.481057
H 1.585214 6.254911 1.270712

$M^{-}(\Pi^{*}).Et_4$
E = -3928.570370
6 -5.002362 -1.180068 0.158239
35 -5.525825 0.589699 0.892040
6 -4.091190 -0.986737 -1.040190
8 -4.536919 -0.951474 -2.193037
7 -2.777121 -0.876504 -0.774069
6 -1.967567 -0.771556 0.457606
6 -0.756370 -0.492669 -0.454007
6 -1.668130 -0.581846 -1.694088
7 -0.073562 0.808504 -0.241622
8 0.481820 1.332901 -1.245741
8 0.168693 1.142415 0.951055
7 0.340127 -1.493314 -0.387990
8 1.022174 -1.668148 -1.435422
8 0.709422 -1.861035 0.761611
1 -4.535511 -1.722469 0.983613
1 -5.928576 -1.660047 -0.159482
1 -1.886550 -1.698639 1.033367
1 -2.234733 0.068177 1.106662
1 -1.776851 0.361986 -2.238311
1 -1.419689 -1.395738 -2.381970
1 1.746999 -3.420058 1.093632
8 2.200957 -4.222082 1.409030
6 2.555083 -5.025250 0.275865
6 3.304316 -6.252673 0.765277
1 1.646415 -5.329766 -0.271307
1 3.184408 -4.443379 -0.417159
1 3.586606 -6.888553 -0.085586
1 4.220494 -5.962271 1.298337
1 2.679088 -6.846366 1.447113
1 2.926519 -1.804803 -1.376420
8 3.895478 -1.842784 -1.472636
6 4.494322 -1.398241 -0.248534
6 6.005042 -1.456478 -0.397039
1 4.173586 -0.367486 -0.022447
1 4.168850 -2.040981 0.586068
1 6.489802 -1.120512 0.530358
1 6.335945 -2.483104 -0.607411
1 6.342372 -0.807568 -1.217533
1 0.986461 3.167276 -1.286959
8 1.186918 4.108911 -1.435595
6 2.598850 4.247383 -1.637869
6 2.917223 5.717183 -1.852326
1 3.145151 3.861722 -0.761420
1 2.915154 3.658825 -2.515823
1 3.995652 5.851690 -2.015587
1 2.383893 6.107651 -2.730421
1 2.623413 6.310828 -0.975239
1 1.809169 1.979493 1.449337
8 2.627829 2.358183 1.818386
6 2.336670 3.669010 2.317431
6 3.613109 4.269831 2.881400
1 1.941889 4.303858 1.506643

1 1.565140 3.612372 3.104218
1 3.415988 5.276031 3.276660
1 4.008441 3.649606 3.698187
1 4.383331 4.349516 2.101441

$M^{-}.Et_4$
E = -3928.561148
C 2.914497 -3.340961 -0.265429
C 2.075406 -2.820478 0.887115
N 1.243858 -1.802618 0.599398
C 1.015365 -0.948420 -0.580502
C 0.081776 -0.083570 0.305493
N -1.426072 -0.244422 -0.151142
O -1.830778 -1.394879 -0.291988
Br 4.611929 -2.315769 -0.367520
O 2.143555 -3.317309 2.017079
C 0.394479 -1.018718 1.504272
N 0.417381 1.288194 0.448355
O 0.737960 1.944044 -0.634597
O 0.067566 1.873938 1.559101
O -2.091812 0.760861 -0.330789
O -0.069393 4.626393 1.809717
C -0.740246 5.292544 0.739771
C -2.223907 4.943085 0.655764
H -0.459038 -1.572707 1.907924
H 0.938000 -0.505512 2.303620
H 0.524830 -1.457859 -1.416244
H 1.892469 -0.389690 -0.920375
H 2.437129 -3.231842 -1.242008
H 3.198464 -4.376550 -0.075168
H -0.025270 3.663528 1.612020
H -0.617288 6.369441 0.925041
H -0.247161 5.065572 -0.220783
H -2.703400 5.510429 -0.156029
H -2.363358 3.872141 0.451404
H -2.734696 5.187690 1.597933
H -3.739978 -2.149193 -0.501314
O -4.553022 -2.675107 -0.529262
C -4.279371 -3.961612 0.046637
C -5.551072 -4.789980 0.001287
H -3.476042 -4.464691 -0.517154
H -3.937555 -3.843965 1.088530
H -5.369864 -5.783390 0.434610
H -6.353322 -4.304246 0.574255
H -5.893259 -4.922027 -1.034676
H -4.014727 0.891415 -1.043064
O -4.903456 1.085585 -1.376259
C -5.832744 0.956822 -0.288524
C -7.226173 1.266636 -0.806182
H -5.794047 -0.066878 0.117933
H -5.563152 1.655319 0.521183
H -7.959335 1.177522 0.007712
H -7.275902 2.289408 -1.205470
H -7.509656 0.566518 -1.604540
H 2.111398 3.052299 -0.496412
O 2.897338 3.644782 -0.496115
C 3.943062 3.009306 -1.233060
C 5.168078 3.909238 -1.231463
H 4.192065 2.033495 -0.780095
H 3.618266 2.817955 -2.271201
H 5.986764 3.439802 -1.795021
H 4.939766 4.878389 -1.697225
H 5.515646 4.091653 -0.204626

[M-NO_2+H]
E = -3103.923247
C 1.983198 0.676494 0.810874
Br 2.538042 -0.899241 -0.263273
C 0.863565 1.413572 0.098949
O 1.089612 2.383920 -0.633262
N -0.382686 0.958349 0.323304
C -1.656922 1.420301 -0.243975
C -2.352220 0.334173 0.612432
C -0.966239 -0.165277 1.082615
N -3.182234 -0.662068 -0.148596
O -3.882686 -0.232710 -1.057933
H -3.015061 0.703677 1.399661
O -3.144833 -1.831047 0.217314
H -1.914021 2.453885 0.011418
H -1.739842 1.269115 -1.326995
H -0.802014 -0.117088 2.164558
H -0.690217 -1.154368 0.699308
H 1.698360 0.286955 1.791279
H 2.860708 1.320413 0.878045

[M-Br]$^-$
E = -734.381527
C 0.655264 -0.022831 -0.002926
N 1.472589 1.246272 -0.153250
N 1.666043 -1.147948 0.143995
O 1.267362 -2.273308 -0.108898
O 2.783036 -0.861769 0.556129
O 1.534002 2.012587 0.795183
O 2.009977 1.408394 -1.241372
C -0.445407 -0.054077 1.077694
C -0.447831 -0.237455 -1.059210
H -0.264870 -1.063365 -1.754328
H -0.659886 0.684878 -1.617350
N -1.390519 -0.512991 0.041631
H -0.271788 -0.744041 1.912299
H -0.648251 0.955476 1.462936
C -2.744222 -0.025739 -0.053341
O -3.214048 -0.018951 -1.243656
C -3.373665 0.342594 1.124155
H -2.880743 0.271661 2.092565
H -4.418719 0.647483 1.099931

[M-Br]
E = -734.230399
C 0.685332 -0.016308 0.003463
N 1.420359 1.317635 0.030268
N 1.752130 -1.095388 -0.003554
O 2.150548 -1.469591 -1.095356
O 2.139276 -1.494006 1.084010
O 0.700983 2.303213 0.050611
O 2.640044 1.312702 0.028279
C -0.417553 -0.161111 1.080113
C -0.401986 -0.126958 -1.094999
H -0.273153 -0.989456 -1.757523
H -0.535175 0.790235 -1.680063
N -1.382405 -0.306660 -0.022096
H -0.297044 -1.047063 1.712228
H -0.547246 0.738760 1.691566
C -2.738880 -0.145176 -0.110468
O -3.281728 -0.032529 -1.226141
C -3.479593 -0.145010 1.135141
H -2.996124 -0.268476 2.102284
H -4.558753 -0.024036 1.088458

$[M-NO_2]^-$ E = -3103.457910
C -1.618696 1.305523 -0.356020
N -0.393695 0.773177 0.289151
C -0.999830 -0.531762 0.677920
C -2.254659 -0.002137 0.036748
H -2.003845 2.218099 0.127078
H -1.488182 1.499593 -1.433128
N -3.459758 -0.491672 -0.097221
O -3.739609 -1.674467 0.365863
O -4.384974 0.199800 -0.693861
H -1.030372 -0.694377 1.767553
H -0.520827 -1.398266 0.194641
C 0.842786 1.285145 0.329440
O 1.130257 2.398977 -0.136074
C 1.895443 0.424310 1.007706
H 2.692487 1.056578 1.400924
H 1.494387 -0.238207 1.777945
Br 2.759853 -0.762712 -0.332592

NO_2
E = -205.105213
N 0.000000 0.000000 0.324830
O -0.000000 1.103173 -0.142113
O -0.000000 -1.103173 -0.142113

EtO^-
E = -154.491929
O 1.311555 -0.122714 -0.000000
C 0.000000 -0.531821 0.000000
H -0.259488 -1.176852 -0.886037
H -0.259488 -1.176852 0.886037
C -1.023588 0.625461 0.000000
H -2.064019 0.253151 0.000000
H -0.883957 1.260213 0.890553
H -0.883957 1.260213 -0.890553

7. References

References

[1] J. Kočišek, B. Sedmidubská, S. Indrajith, M. Fárník, J. Fedor, *The Journal of Physical Chemistry B* **2018**, *122*.

[2] M. Fárník, J. Fedor, J. Kočišek, J. Lengyel, E. Pluhařová, V. Poterya, A. Pysanenko, *Phys. Chem. Chem. Phys.* **2021**, *23*, 3195.

[3] J. Kočišek, A. Pysanenko, M. Fárník, J. Fedor, *The Journal of Physical Chemistry Letters* **2016**, *7*, 3401–3405.

[4] R. Dressler, M. Allan, *Chemical Physics* **1985**, *92*, 449.

[5] J. Belloni, H. Monard, F. Gobert, J.-P. Larbre, A. Demarque, V. De Waele, I. Lampre, J.-L. Marignier, M. Mostafavi, J. Bourdon, M. Bernard, H. Borie, T. Garvey, B. Jacquemard, B. Leblond, P. Lepercq, M. Omeich, M. Roch, J. Rodier, R. Roux, *Nuclear Instruments and Methods in Physics Research Section A: Accelerators, Spectrometers, Detectors and Associated Equipment* **2005**, *539*, 527.

[6] S. Denisov, ELYSE platform.

[7] M. J. Frisch, G. W. Trucks, H. B. Schlegel, G. E. Scuseria, M. A. Robb, J. R. Cheeseman, G. Scalmani, V. Barone, G. A. Petersson, H. Nakatsuji, X. Li, M. Caricato, A. V. Marenich, J. Bloino, B. G. Janesko, R. Gomperts, B. Mennucci, H. P. Hratchian, J. V. Ortiz, A. F. Izmaylov, J. L. Sonnenberg, D. Williams-Young, F. Ding, F. Lipparini, F. Egidi, J. Goings, B. Peng, A. Petrone, T. Henderson, D. Ranasinghe, V. G. Zakrzewski, J. Gao, N. Rega, G. Zheng, W. Liang, M. Hada, M. Ehara, K. Toyota, R. Fukuda, J. Hasegawa, M. Ishida, T. Nakajima, Y. Honda, O. Kitao, H. Nakai, T. Vreven, K. Throssell, J. A. Montgomery, Jr., J. E. Peralta, F. Ogliaro, M. J. Bearpark, J. J. Heyd, E. N. Brothers, K. N. Kudin, V. N. Staroverov, T. A. Keith, R. Kobayashi, J. Normand, K. Raghavachari, A. P. Rendell, J. C. Burant, S. S. Iyengar, J. Tomasi, M. Cossi, J. M. Millam, M. Klene, C. Adamo, R. Cammi, J. W. Ochterski, R. L. Martin, K. Morokuma, O. Farkas, J. B. Foresman, D. J. Fox, Gaussian~16 Revision A.03 **2016**, gaussian Inc. Wallingford CT.

[8] K. Iwamatsu, G. Holmbeck, R. Gakhar, P. Halstenberg, B. Layne, S. Pimblott, J. Wishart, *Physical Chemistry Chemical Physics* **2022**, *24*, 25088.

[9] D. Dobrovolskii, M. Mostafavi, S. Denisov, *Physical Chemistry Chemical Physics* **2023**, *25*, 15916.

[10] M. Orzol, I. Martin, J. Kocisek, I. Dabkowska, J. Langer, E. Illenberger, *Phys. Chem. Chem. Phys.* **2007**, *9*, 3424.

[11] A. Paul, S. Ghosh, D. Nandi, *Phys. Chem. Chem. Phys.* **2023**, *25*, 28263.

[12] M. Kaneda, M. Shimizu, T. Hayakawa, Y. Iriki, H. Tsuchida, A. Itoh, *The Journal of Chemical Physics* **2010**, *132*, 144502.

[13] R. Meissner, J. Kočišek, L. Feketeová, J. Fedor, M. Fárník, P. Limáo-Vieira, E. Illenberger, S. Denifl, *Nature Communications* **2019**, *10*, 2388.
